# A prospective study of biomarker-guided chemotherapy in patients with non-small cell lung cancer

**DOI:** 10.1007/s00280-014-2513-x

**Published:** 2014-08-14

**Authors:** Qiang Zhang, Xiaoli Zhu, Li Zhang, Siqing Sun, Jing Huang, Yong Lin

**Affiliations:** Department of Pulmonary Medicine, Zhongda Hospital Affiliated to Southeast University, Dingjiaqiao No. 87, Nanjing, 210009 People’s Republic of China

**Keywords:** Chemotherapy, Non-small cell lung cancer, Molecular biomarkers, Efficacy

## Abstract

**Purpose:**

To assess the therapeutic value of biomarker-guided chemotherapy in patients with advanced non-small cell lung cancer (NSCLC).

**Methods:**

Eighty-five NSCLC patients at stage IIIb or IV were divided into two groups based on the feasibility of biomarker analysis. Group A included patients with biomarker data (*n* = 41); Group B were patients without biomarker results (*n* = 44). Tumor samples obtained by fiberoptic bronchoscopy and computerized tomography-guided needle biopsy were analyzed by immunohistochemistry for intratumoral level of excision repair cross-complementing gene 1 (ERCC1), ribonucleotide reductase M1 (RRM1), and β-tubulin III. Chemotherapy regimens in Group A were determined according to the status of molecular signatures, whereas a standard gemcitabine plus cisplatin regimen was used for Group B. Tumor response, patient survival, and adverse effects were monitored for both groups.

**Results:**

The overall response rate, defined as complete response plus partial response, was 56.1 % for Group A, significantly higher than that in Group B (31.8 %; *P* = 0.024). The median progression-free survival (PFS) time was 5.2 months for Group A, significantly longer than that of Group B (4.1 months; *P* = 0.026). The 1-year survival rate of Group A was 65.9 %, significantly higher than that of Group B (40.9 %; *P* = 0.021), whereas the median overall survival times were 13.5 versus 12.5 months for Groups A and B, respectively (*P* = 0.483). The adverse effects in the two groups were essentially the same.

**Conclusions:**

Biomarker-tailored chemotherapy based on ERCC1, RRM1, and β-tubulin III expression showed significantly increased response rate, median PFS time, and 1-year survival rate in patients with NSCLC.

## Introduction

Lung cancer is one of the leading causes of cancer-related mortality for both men and women worldwide [1]. Non-small cell lung cancer (NSCLC) accounts for approximately 75–80 % of all lung cancer cases. Nearly two-thirds of NSCLC patients have unresectable advanced diseases upon diagnosis. Combined modality treatment has become the standard of care for cancer therapy in the past few years, and chemotherapy is an integral part of the treatment for lung cancer [2]. For the patients with advanced NSCLC, chemotherapy prolongs survival and improves quality of life and platinum-based chemotherapy has been accepted as a standard therapy [3]. However, for those patients, the 2-year overall survival (OS) rate remains <15 %. Despite tremendous efforts devoted to develop monotherapy or combinational chemotherapy regimens, therapeutic outcomes are still poor [4].

Recently, it has been increasingly suggested that tailored individual chemotherapy based on molecular biomarkers represents a novel avenue for NSCLC treatment [5, 6]. Indeed, biomarker-based molecularly targeted therapy has made an unprecedented progress in the treatment for NSCLC in the past decade. A good example is the application of epidermal growth factor receptor (EGFR) inhibitors to a subset of patients bearing certain mutations, which results in a favorable response [5]. Likewise, excision repair cross-complementing gene 1 (ERCC1), ribonucleotide reductase M1 (RRM1), and β-tubulin III statuses have been reported to correlate with the therapeutic efficacy of platinum, gemcitabine, and docetaxel, respectively [6–9]. While biomarker-guided chemotherapy shows potentials in benefiting patients, most evidences came from retrospective studies and are often limited to a single molecular signature [10–12]. It has been increasingly recognized that a single molecule may not be able to precisely predict the patient’s response, and a cluster of molecular biomarkers would be more appropriate to guide clinical decision-making.

Platinum, gemcitabine, and docetaxel represent the most important chemotherapeutic agents used in the treatment of advanced NSCLC [2]. Recently, a growing number of studies aimed to identify molecular signatures that can predict the clinical outcomes of these agents. Among the suggested biomarkers, ERCC1 is well-studied. ERCC1 is a critical component of nucleotide excision repair, a primary DNA repair mechanism that removes platinum–DNA adducts from genomic DNA [7]. It has been reported that ERCC1-negative tumors have a better response to adjuvant cisplatin-based chemotherapy [8], whereas high expression of ERCC1 results in drug resistance [9]. Both retrospective [10] and prospective [11] studies aiming to assess the predictive utility of ERCC1 status for platinum-based chemotherapy in NSCLC have revealed improved clinical outcomes by integrating patient’s ERCC1 status in developing individualized therapy [12, 13]. In terms of gemcitabine-based therapy, RRM1, which catalyzes a rate-limiting step in the production of deoxyribonucleotides required for DNA synthesis, has been strongly suggested as a candidate biomarker [14]. An increased level of RRM1 enables cancer cells to more efficiently repair DNA damage caused by chemotherapy, resulting in resistance to gemcitabine. In fact, RRM1 is believed to be the predominant cellular determinant of the efficacy of the nucleoside analogue gemcitabine [15]. For instance, a meta-analysis of Gong et al. [16] has shown that RRM1-low or RRM1-negative advanced NSCLC is associated with a higher response rate to gemcitabine-containing regimen and a better prognosis. Taxanes exert their anti-tumor effects by binding to and stabilizing intracellular microtubules [17]. Recent studies have revealed that the expression of β-tubulin III is associated with the tumor sensitivity to docetaxel treatment [18]. Patients with lower levels of β-tubulin III are more sensitive to docetaxel treatment [19, 20], while β-tubulin III high expression represents an independent risk factor for the poor progression-free survival (PFS) of cancer patients receiving such treatment [21]. Collectively, these findings demonstrate that the β-tubulin III mRNA level can be used as an independent predictive biomarker for the outcome of the paclitaxel-/vinorelbine-based chemotherapy [22].

In the present study, we attempted to integrate ERCC1, RRM1, and β-tubulin III expression levels into the decision-making of chemotherapy for NSCLC. It was hoped that this prospective, feasibility study would help assess the therapeutic values of the molecular biomarker-guided chemotherapy for the treatment of advanced NSCLC.

## Patients and methods

### Patients

A total of 85 advanced NSCLC patients admitted at our hospital (Zhongda Hospital Affiliated to Southeast University, Nanjing, People’s Republic of China) between January 2007 and August 2011 were recruited into this study. The following inclusion criteria were used: (1) pathological- and radiological-based diagnosis of stage IIIB or IV, inoperable NSCLC; (2) measurable tumors using computerized tomography (CT) scanning; (3) Eastern Cooperative Oncology Group (ECOG) performance status between 0 and 2; (4) predicted survival time ≥3 months; and (5) signed informed consent to participate in the study.

Patients were excluded if any of the following criteria was met: (1) ECOG performance status >2; (2) severe complications, including cardiovascular and pulmonary diseases, bone marrow suppression, liver and renal dysfunctions, and/or organ failures; (3) brain metastasis; (4) chemotherapy history; (5) predicted survival time <3 months; and (6) unwillingness to receive chemotherapy.

### Clinical trial design

The trial was designed to prospectively assess the therapeutic values of intratumoral molecular analysis in guiding chemotherapy options for patients with advanced NSCLC. Patients with sufficient tumor tissue amounts obtained by either fiberoptic bronchoscopy or CT-guided needle biopsy for molecular probing were assigned into Group A, and the rest of patients were assigned into Group B as controls. Intratumoral expression levels of ERCC1, RRM1, and β-tubulin III were examined by immunohistochemistry (IHC) analysis, which was used for determining the chemotherapy regimens for each patient. Therapy regimens for patients in Group B were based on the National Comprehensive Cancer Network Clinical Practice Guidelines for NSCLC. They were planned to be treated with standard first-line gemcitabine–cisplatin regimens for 4–6 cycles (21 days per cycle). In case of disease progression or severe adverse effects, regimens were adjusted: Patients received follow-up treatment using single-agent or second-line treatment until death or the end of the study.

The study protocol was reviewed and approved by the Ethical Committee of Zhongda Hospital Affiliated to Southeast University, Nanjing, People’s Republic of China. As aforementioned, each of the patients signed an informed consent before entry into the study.

### Specimen collection and analysis

Tumor specimens from patients were initially obtained by using fiberoptic bronchoscopy. However, for tumors located beyond the coverage of fiberoptic bronchoscopy, mostly in peripheral pulmonary areas, CT-guided needle biopsy was applied. Tissues were fixed in 10 % neutral-buffered formalin, embedded in paraffin wax, and then sectioned to 2-µm-thick slides for the analysis for ERCC1, RRM1, or β-tubulin III expression using IHC with the PO-7000 commercial kit (ZSGB-Bio, Beijing, People’s Republic of China) following the manufacturer’s instructions. Primary antibodies were as follows: mouse anti-ERCC1 antibody (Cat# 12A00409; ZSGB-Bio, Beijing, People’s Republic of China), rabbit anti-RRM1 antibody (Cat# 60073-1-lg; BJGB-Bio, Beijing, People’s Republic of China), and mouse anti-III β-tubulin antibody (Cat#1226P1011B; BJGB-Bio, Beijing, People’s Republic of China).

Semiquantitative analysis of the IHC staining was performed independently by two experienced pathologists. The intensity of the staining was graded by the following scale: 0 = no staining, + = weak staining, and ++ = strong staining. Staining in human fetal cells, which were primary cells derived from human specimen and validated pathologically in our hospital (++) as well as human tonsil cells (++), and human brain cells (++), was used as positive controls for scoring the ERCC1, RRM1, and β-tubulin III staining, respectively.

### Chemotherapy regimens

Patients in Group A received different chemotherapy regimens according to their ERCC1, RRM1, and β-tubulin III expression statuses. Patients with ERCC1-negative or ERCC1-low were given platinum-based doublets (cisplatin or carboplatin in combination with docetaxel, paclitaxel, vinorelbine, gemcitabine, or pemetrexed); RRM1-negative or RRM1-low received either gemcitabine single-agent or gemcitabine-based doublets; and patients with high β-tubulin III expression avoided taxanes. As pemetrexed is not recommended for treatment of squamous carcinoma, those patients with high expression of RRM1 and β-tubulin III were not included in this study. Based on these principles, patients in Group A were divided into seven subgroups receiving various regimens (Table 1). All patients in Group B were treated with gemcitabine plus cisplatin in every 21-day cycle.

**Table 1:** Treatment regimens for patients in Group A

Biomarkers	RRM1−	β-Tubulin III−	β-Tubulin III+
ERCC−	Gemcitabine + cisplatin	–	–
ERCC − plus RRM1+	–	Paclitaxel + carboplatin/docetaxel + cisplatin	Pemetrexed + cisplatin
ERCC + plus RRM1−	–	Gemcitabine + paclitaxel	Gemcitabine
ERCC + plus RRM1+	–	Paclitaxel + docetaxel	Pemetrexed

Therapy regimen: gemcitabine: 1,000 mg/m^2^, days 1 and 8; cisplatin: 75 mg/m^2^ within 3 days; paclitaxel: 135 mg/m^2^, day 1; carboplatin: area under the time–concentration curve = 6, day 1; docetaxel: 75 mg/m^2^, day 1; pemetrexed: 500 mg/m^2^, day 1

− = Low expression; + = High expression

Patients in both groups were given routine anti-vomiting medications prior to chemotherapy. For those receiving regimens containing cisplatin, adequate hydration was given. During the treatment, routine blood tests and liver and renal functions were monitored.

### Efficacy assessment

Prior to chemotherapy, all patients provided their treatment history and underwent a series of medical examinations, including pathological examination, CT scan of chest (upper abdomen and adrenal glands), emission CT bone scan, brain CT, or magnetic resonance imaging, complete blood cell counts, platelet counts, and biochemical analysis for liver and renal functions. All tests were repeated every two cycles of treatment. Tumor response was assessed according to the RECIST 1.1 criteria [23] and categorized as complete response (CR), partial response (PR), stable disease (SD), and progressive disease (PD). Response rate (%) was defined by following formula: (CR + PR)/all treated patients × 100 %. Disease control rate (DCR) (%) was defined by following formula: (CR + PR + SD)/all treated patients × 100 %.

Survival analyses were performed on an intent-to-treat basis. OS was calculated from the date of assignment to either the date of death or last clinical follow-up, whichever occurred first. PFS was the time interval between the dates of first treatment and either disease progression or death, whichever occurred first. Adverse effects caused by chemotherapy were scored by 0–IV degrees based on the WHO standard.

### Statistical analysis

Patients’ characteristics were described as median (interquartile range—IQR = 25–75 %) or frequencies (%). Continuous variables were compared using the Kruskal–Wallis test. Categorical variables were compared using the *χ* ^2^ test. Kaplan–Meier survival curves were compared by using the log-rank test. *P* values <0.05 were considered statistically significant. Data were analyzed using the SPSS v17.0 statistical software package (SPSS, Inc., Chicago, IL, USA).

## Results

### Patients’ characteristics

**Fig. 1:**
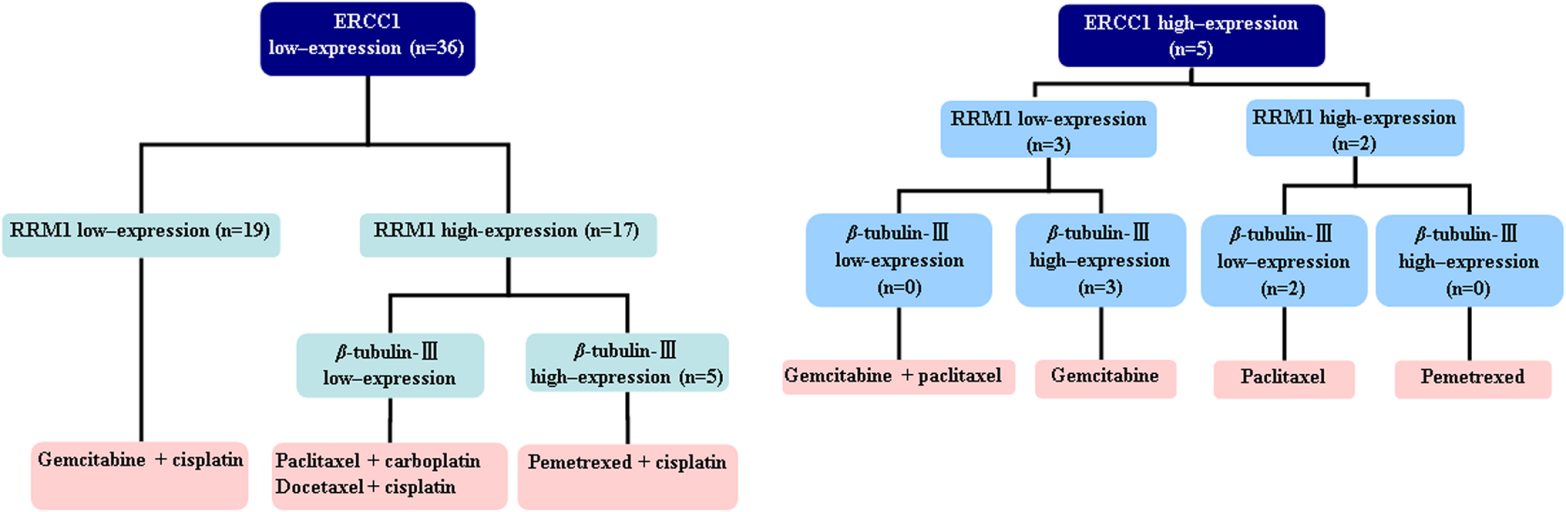
A workflow chart showing a molecular signature tailored chemotherapy trial for the treatment for NSCLC. Flowchart and treatment algorithm used for selection of tailored chemotherapy based on molecular signature. Patients with ERCC1-negative or ERCC1-low were given platinum-based doublets; RRM1-negative or RRM1-low received either gemcitabine single agent or gemcitabine-based doublets; and patients with high β-tubulin III expression avoided taxanes

From January 2007 to August 2011, 85 patients were registered into the trial. Biopsy was obtained from each patient. Among them, 31 had CT-guided lung biopsies, 50 had bronchoscopy-guided lung biopsies, and 4 had biopsies from organs other than lung. All patients completed the study, and 41 of them, whose biopsies were suitable for IHC, were subjected to measurements of ERCC1, RRM1, and β-tubulin III. Patients’ characteristics in both groups were compared, and there were no significant difference in patients number, gender, age, or ECOG performance status scores (*P* > 0.05; Table 2) (Fig. 1).

**Table 2:** Patients’ characteristics

	Total	Group A	Group B	*P*
Patients’ number (%)	85	41 (48.2)	44 (51.8)	–
Gender				
Male (%)	54	25 (46.3)	29 (53.7)	0.637
Female (%)	31	16 (51.6)	15 (48.4)	
Age				
Range	29–85	29–78	34–85	–
Medians	65	64	62	
ECOG performance status 0/1/2	–	11/24/6	9/26/9	0.679
Pathological types				
Squamous cell carcinoma (%)	36	17 (47.2)	19 (52.8)	0.873
Adenocarcinoma (%)	49	24 (49.0)	25 (51.0)	
Stages				
IIIB (%)	37	19 (51.4)	18 (48.6)	0.614
IV (%)	48	22 (45.8)	26 (54.2)	
Chemotherapy regimens				
Gemcitabine +cisplatin (%)	–	19 (46.3)	44 (100.0)	–
Paclitaxel + carboplatin (%)	–	6 (14.6)	–	
Docetaxel + cisplatin (%)	–	6 (14.6)	–	
Pemetrexed + cisplatin (%)	–	5 (12.2)	–	
Gemcitabine (%)	–	3 (7.3)	–	
Paclitaxel (%)	–	2 (4.9)	–	
Gemcitabine + paclitaxel (%)	–	0 (0.0)	–	
Pemetrexed (%)	–	0 (0.0)	–	

Group A: inter-quartile range 25–75 % of age between 56 and 70.5; Group B: inter-quartile range 25–75 % of age between 53.25 and 70

### Efficacy

We firstly compared the response rates of patients in both groups. Among those in Group A, treatment responses were as follows: CR in none patient, PR in 23 patients = 56.1 % (95 % CI 39.1–70.7 %), SD in 13 patients = 31.7 % (95 % CI 17.1–46.3 %), and PD in five patients = 12.2 % (95 % CI 2.4–22.0 %). The overall response rate (CR + PR) was 56.1 %. In contrast, for patients in Group B, the responses included CR in none patient, PR in 14 patients = 31.8 % (95 % CI 18.2–45.5 %), SD in 20 patients = 45.5 % (95 % CI 29.5–61.3 %), and PD in ten patients = 11.4 % (95 % CI 2.3–20.5 %). The overall response rate in Group B was 31.8 %, significantly lower than that in Group A (*P* = 0.024).

**Fig. 2:**
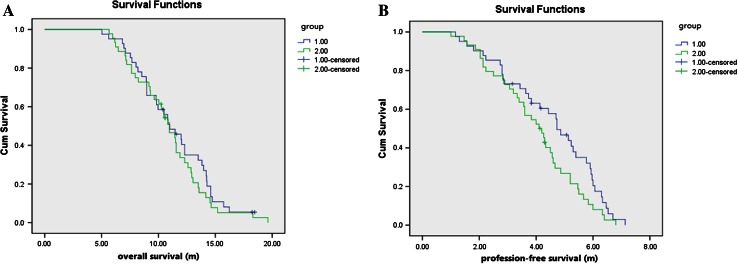
Comparison of therapeutic efficacy. **a** OS curves. **b** PFS estimates by treatment arm. *Blue line* Group A chemotherapy regimens were determined according to the patient’s molecular signatures; *green line* Group B treatment with gemcitabine plus cisplatin regimen. The differences in median OS were not statistically significant (log-rank test *P* = 0.483). The median PFS time of Group A is significantly longer than Group B (log-rank test *P* = 0.026)

We next compared survivals of patients in both groups. PFS and OS curves are shown in Fig. 2. Median PFS times were 5.2 months (IQR = 2.85–5.90 months) for Group A versus 4.1 months (IQR = 2.85–4.82 months) for Group B, showing significant differences between the two groups (*P* = 0.026). It was also noticed that 1-year survival rate in Group A was higher than that in Group B (65.9 vs. 40.9 %, respectively; *P* = 0.021). However, the median survival times of both groups were very similar (13.5 months—IQR = 8.97–15.57 months) for Group A versus 12.5 months (IQR = 7.72–17.34 months) for Group B; *P* = 0.483). There was no difference in DCR rate between the two groups 87.8 versus 77.3 % (*P* = 0.203) (Table 3).

**Table 3:** Therapeutic efficacy assessment

Outcome	Group A (*n* = 41)	Group B (*n* = 44)	*χ* ^2^	*P*
CR	0	0	–	–
PR	23	14	–	–
SD	13	20	–	–
PD	5	10	–	–
ND	0	0	–	–
OR (%)	56.1	31.8	5.09	0.024
DCR (%)	87.8	77.3	1.62	0.203
Median survival time (month)	13.5	12.5	0.513	0.483
Median PFS time (month)	5.2	4.1	5.498	0.026

*CR* Complete response, *PR* partial response, *SD* stable disease, *PD* progressive disease, *ND* not determined, *OR* overall response rate, *DCT* disease control rates

### Adverse effects

At the time of assessment, patients in Group A had received 4.05 cycles of treatment whereas those in Group B had received 4.23 cycles. Bone marrow suppression, gastrointestinal reactions, and liver and renal dysfunctions represented the most common adverse effects caused by chemotherapy. We next compared the adverse effects resulting from chemotherapy in the two groups (Table 4). Toxicities greater than grade III were very similar in the two groups (*P* = 0.431 for bone marrow suppression; *P* = 0.911 for nausea and vomiting; *P* = 0.564 for liver and renal dysfunctions). Moreover, methods for toxicity management, such as anti-vomiting medications, were effective in both groups.

**Table 4:** Adverse effect assessment

	Group A			Group B		
	0	I + II	III + IV	0	I + II	III + IV
Bone marrow suppression (%)*	13 (31.7)	25 (61.0)	3 (7.3)	11 (25.0)	26 (59.1)	7 (15.9)
Nausea plus vomiting (%)^#^	15 (36.6)	25 (61.0)	1 (2.4)	18 (40.9)	25 (56.8)	1 (2.3)
Liver and renal dysfunctions (%)^†^	33 (80.5)	7 (17.1)	1 (2.4)	31 (70.5)	10 (22.7)	3 (6.8)

* *χ* ^2^ = 1.683, *P* = 0.431

^#^Fisher’s exact, *P* = 0.911

^†^Fisher’s exact, *P* = 0.564

## Discussion

Currently, therapy with platinum-based doublets is the standard care for first-line treatment of patients with advanced NSCLC and has achieved a good performance status (0–1) [3]. However, the selection of chemotherapeutic agents is generally based on convenience, side effect profiles, and the physician’s experience. The individualized chemotherapy is lagging far behind molecularly targeted therapy, which has validated molecular signatures in NSCLC and clearly defined target patient populations. In spite of the increasing number of clinical studies to explore therapeutic opportunities of individualized chemotherapy based on a large number of patients, such as BATTLE trial [24], it remains unclear how to tailor regimens for individual patient to further improve the therapeutic efficacy.

We have noticed that an increasing number of molecules have been suggested to correlate with the chemo-sensitivity in clinical studies. These candidate biomarkers mostly stem from the growing understanding of molecular action mechanisms for these agents. It is conceivable that stratifying the patients based on these molecular signatures will improve the effectiveness of chemotherapy and/or reduce side effects. However, prospective studies in this regard are rare, and, among the limited studies, findings are often in discrepancy. Our prospective study aimed to assess the predictive utility of ERCC1, RRM1, and β-tubulin III expression in chemotherapy of NSCLC. By simultaneously integrating the expression status of three candidate biomarkers into the regimen selection, we compared the response rates, PFS, OS, and toxicities between patients treated with biomarker-guided therapy and those treated with standard gemcitabine/cisplatin regimen, a widely used combination in any stage of NSCLC and with advantages in improving the OS or PFS compared to other platinum-based regimens, suggested by a meta-analysis [25]. We observed the significantly improved response rate, PFS time, and 1-year survival rate, but no change in the OS rate or DCT. However, we noticed that a recent prospective study by Bepler et al. [26] did not observe a survival or response rate from individualized therapy using ERCC1 and RRM1 expression as molecular signatures. We speculate that the difference may, at least partially, stem from the different approaches used for measuring expression of molecular signatures. In their study, Bepler et al. [26] used RT-PCR instead of IHC to measure the intratumoral expression of ERCC1 and RRM1, which may not ensure the detection of functional proteins. Also, it was a multicenter study, and criteria between the centers might vary. The authors have claimed that their finding is false negative and believed that protein expression analysis for therapeutic decision-making is feasible in newly diagnosed patients with advanced-stage NSCLC [26].

In the present study, we also compared the adverse effects after chemotherapy in the two groups. Common adverse effects, like bone marrow suppression and gastrointestinal reactions, were observed in both groups and most were grade I or II toxicities. There was no statistic difference between the two groups, suggesting that chance of increased adverse effects resulted from individualized chemotherapy is relatively small, further strengthening its clinical potential.

Meanwhile, it is important to mention that our study has its limitations in patients grouping. It is a feasibility study rather than a randomized study. Although we did not observe significant differences in patient number, gender, age, or ECOG performance status scores between the two groups, a randomized control trial is needed to verify our results in the future.

The integration of molecular biomarkers into the clinical decision-making largely relies on the molecular profiling in cancer tissues. Lately, multiple approaches are being developed [8]. Currently, IHC analysis [10], RT-PCR [21], and quantitative analysis of in situ protein [27] have been performed in retrospective studies. However, it still requires more studies to conclude on an appropriate approach for clinical applications [28]. IHC provides information on the protein expression level and its localization, which has become the standard in situ assay to assess protein expression. IHC may prove to be the most attractive option based on the relative uniform results, few limitations, and a broad clinical applicability. However, IHC is semiquantitative, subjective, and may be affected by a range of poorly controlled variables. For example, Friboulet et al. [29] have reported that IHC analysis with the use of currently available ERCC1 antibodies does not specifically detect the unique functional ERCC1 isoform, which may limit the utility of ERCC1 in predicting the patients’ responsiveness to therapy. Instead, they reached their conclusion based on the analysis of isolated cells. Likewise, Toffart et al. [30] have analyzed up to eight molecules, such as ERCC1, BRCA1, and TUBB3, in small size of non-surgical biopsies using IHC and failed to reveal the associated therapeutic efficacy in terms of DCR, PFS, or OS. We included the biomarkers into regimen assignments, which may account for the different conclusions between our studies and Toffart et al.’s [30] study. As indicated by these authors, also the small size of non-surgical biopsies often left no slides for IHC studies. That is indeed the reason which restricted us from a randomized design and instead subgrouping the patients according to the feasibility of the IHC.

In oncology clinic, quality assessment tools for examining prognostic and predictive biomarkers are still lacking. For prospective studies, the ability to carry out molecular detection with a small amount of tissues, like biopsies, is essential. Attempts to detect gene expression in bronchoscopic or fine needle aspiration biopsies have emerged lately and not yet been applied broadly. Encouragingly, several recent studies have suggested that the limited amount of bronchoscopic or fine needle aspiration biopsies would allow establishing gene profiling and molecular tumor classification and might become a powerful adjunct for the daily clinical practice [31, 32]. Recently, Suwinski et al. [33] have successfully integrated multiple molecular signatures from bronchoscopic biopsies into predicting clinical outcomes. In agreement with these findings, our study has shown that biopsies from 41 of the 85 patients included were eligible for IHC-based gene expression detection, but its potential for a broad application needs to be examined in future studies.

In conclusion, our prospective study applied IHC to measure ERCC1, RRM1, and β-tubulin III expression in biopsies, and assessed the potential benefits of the three molecular biomarker-guided chemotherapy and the adverse effects in the treatment for NSCLC. Our results have demonstrated that tailored chemotherapy based on ERCC1, RRM1, and β-tubulin III expression showed significantly increased response rate, median PFS time, and 1-year survival rate in patients with NSCLC, without an increase in adverse effects.
